# Functionally Graded Scaffolds with Programmable Pore Size Distribution Based on Triply Periodic Minimal Surface Fabricated by Selective Laser Melting

**DOI:** 10.3390/ma13215046

**Published:** 2020-11-09

**Authors:** Xueyong Zhou, Yuan Jin, Jianke Du

**Affiliations:** 1 School of Mechanical Engineering and Mechanics, Ningbo University, Ningbo 315211, China; 1811081107@nbu.edu.cn; 2 State Key Laboratory of Fluid Power and Mechatronic Systems, School of Mechanical Engineering, Zhejiang University, Hangzhou 310027, China

**Keywords:** functionally graded scaffold, triply periodic minimal surfaces, programmable pore size distribution, mechanical property, selective laser melting

## Abstract

Functional graded materials are gaining increasing attention in tissue engineering (TE) due to their superior mechanical properties and high biocompatibility. Triply periodic minimal surface (TPMS) has the capability to produce smooth surfaces and interconnectivity, which are very essential for bone scaffolds. To further enhance the versatility of TPMS, a parametric design method for functionally graded scaffold (FGS) with programmable pore size distribution is proposed in this study. Combining the relative density and unit cell size, the effect of design parameters on the pore size was also considered to effectively govern the distribution of pores in generating FGS. We made use of Gyroid to generate different types of FGS, which were then fabricated using selective laser melting (SLM), followed by investigation and comparison of their structural characteristics and mechanical properties. Their morphological features could be effectively controlled, indicating that TPMS was an effective way to achieve functional gradients which had bone-mimicking architectures. In terms of mechanical performance, the proposed FGS could achieve similar mechanical response under compression tests compared to the reference FGS with the same range of density gradient. The proposed method with control over pore size allows for effectively generating porous scaffolds with tailored properties which are potentially adopted in various fields.

## 1. Introduction

Scaffolds that are commonly fabricated using metallic materials and bioactive glasses have been widely used for rehabilitative therapy of segmental bone defects [1,2,3,4]. The mechanical properties of scaffolds are expected to match that of natural bone tissue for transferring stress adequately [5,6]. The mechanical strength of bioactive glass scaffolds can be modulated from many aspects, including chemical component adjustment, processing technique, and porosity [7]. However, the elastic moduli of metallic materials are significantly higher, resulting in the surrounding bone tissue not being able to bear adequate load and beginning to degenerate since the osteocytes do not have adequate stress stimulation. This issue caused by the mismatch in the elastic modulus of the scaffold and the bone is known as the stress shielding effect [8,9]. To avoid this issue, the scaffold is generally designed as a porous structure to reduce the elastic modulus to the level of human bones [10,11,12]. Moreover, the distributed pores within the scaffold can effectively strengthen contact between the bone and the scaffold by providing the necessary space for osteocytes and other cells [13,14]. Therefore, scaffold design plays an important role in improving the properties of fabricated porous scaffolds.

A triply periodic minimal surface (TPMS) is a 3D surface with a mean curvature of zero and periodic structures in three coordinate directions and is implicitly expressed using mathematical equations [15,16]. The function-defined surface can be directly enclosed to generate network-based structures or thickened to create sheet-based structures [17]. Porous scaffolds based on TPMS possess various excellent properties, such as continuous and smooth surface, interconnected network, and accurate porosity control [18,19,20,21]. There have been a number of studies concentrating on the performance of TPMS scaffolds, such as surface curvature [19], fatigue behavior [22], and biomechanical influence [23]. These studies indicate that TPMS scaffolds are not only conducive biologically to cell penetration and nutrient diffusion [24] but also beneficial mechanically for mimicking the elastic modulus and strength of bone tissue [25]. Thanks to the development of additive manufacturing (AM), TPMS scaffolds with complex inner structures can be fabricated with high precision, reproducibility, and reasonable cost [26,27,28]. As a result, the TPMS scaffolds can be designed with increasing complexity at both the macro-scale (target specific geometry) and micro-scale (porous topology), offering feasibility for designing functionally graded scaffold (FGS).

Inspired by graded structures in nature and their outperformed properties including high porosity, specific stiffness, energy absorption, and tissue friendly, FGS has drawn increasing attention from different fields [5,29,30,31,32]. The combination of mechanical and biological properties with grading distribution can better match biomechanical properties using local manipulation of the internal pore architectures and can meet the biological requirements for bone tissue regeneration as well. Besides, another advantage of FGS is the possibility to optimize the biological and mechanical performance by properly tuning design parameters to control the spatial distribution of pores in different sizes [33]. The graded porous structures are expected to exhibit similar morphology and mechanical properties with natural bones; multiple methodologies have been proposed for the designing process according to the required biological and mechanical properties [34,35]. Both network and sheet-based TPMS scaffolds can be used to construct FGS by continuously changing several morphological parameters, such as pore size, porosity, and strut thickness.

The mathematics-based modeling approach for the TPMS scaffold enables the generation of FGS with different pore geometries and architectural features readily [36]. There are mainly three methodologies to construct FGS using TPMS structures. First, a gradient in relative density can be achieved by defining the porous domain of unit cells as a function of location vector. This strategy has been widely used for obtaining gradients of biological and mechanical properties [35,37,38]. Afshar et al. [37] chose two different TPMSs (P and D surfaces) to construct linearly graded porosity scaffolds to explore their mechanical responses for stretching and bending dominated deformations compared to their corresponding uniform porous structures. Li et al. [39] tried to achieve better structural or thermal performances by controlling the relative density distribution. Second, grading cell types can be generated with a transition between different cell types by properly selecting the basic substructures and by arranging the control points [40]. Yang et al. adopted this method to generate graded porosity based on micro-CT data [41] and to construct composite porous scaffolds [42] and stochastic porous structures [43]. Third, the gradient in cell size is commonly observed within human bone and can be accomplished by continuously varying the coefficients that determine the cell size in the implicit functions [44]. Al-Ketan et al. investigated the mechanical properties numerically and experimentally for sheet-based TPMS scaffolds with grading in cell size, uncovering that testing along one perpendicular direction exhibits higher Young’s moduli values than the other perpendicular direction and the parallel direction [45].

Clearly, gradients in the relative density, cell type, and cell size of TPMS FGS can be considered effective strategies to optimize the overall performance as well as to enhance the controllability over their physical, mechanical, and geometrical properties. Various applications with grading properties can be accomplished with parametric design on parameters, as mentioned above [45]. However, an issue behind all of these current methods for constructing TPMS-based FGS is that the pore size would change accordingly when one of design parameters varies (relative density, cell type, and cell size). Specifically, since the cell size or the relative density is commonly kept constant for the gradients in relative density or the gradients in cell size, the obtained pore size distribution is directly determined with consideration of only one design parameter and it cannot be modulated further.

Various studies have been conducted to investigate how the pore sizes of porous scaffolds affect their biological performances, and the results revealed that the ability of scaffolds for cells to grow and proliferate is pore size-related [46,47,48,49]. Therefore, the pore size should be fully considered in the scaffold design to achieve graded scaffolds similar to natural bones. As for an FGS designed based on TPMS, the pore size distribution is also required to be programmable to enhance the versatility of the fabricated scaffolds.

In this work, a type of FGS is proposed by changing the spatial distribution of pores to achieve a gradient of relative density, while the pore size is also programmable within the whole scaffold. To verify the feasibility and effectiveness of the proposed method, a typical FGS with constant pore size is adopted in this work for investigation. It should be noted that different gradients on the pore size can be generated using the proposed method. It is noteworthy that both network and sheet-based TPMS structures will be discussed here since they can be both tuned to design scaffolds with biological and mechanical properties similar to that of natural human bones [50]. On the other hand, Gyroid is a widely used TPMS structure and much more suitable in biological contexts [17], so all the samples in this study are designed based on the Gyroid unit cell and then fabricated using Ti6Al4V, for which excellent mechanical properties and biocompatibility have been extensively studied [14,51]. In Section 2, the design and modeling of the proposed FGS is described. Section 3 illustrates the process of fabrication, characterization, and mechanical evaluation. Section 4 presents the observed and recorded results before concluding by summarizing the main contributions of this work in Section 5.

## 2. Design and Modeling of TPMS-Based FGS

### 2.1. TPMS-Based Scaffolds

**Figure 1:**
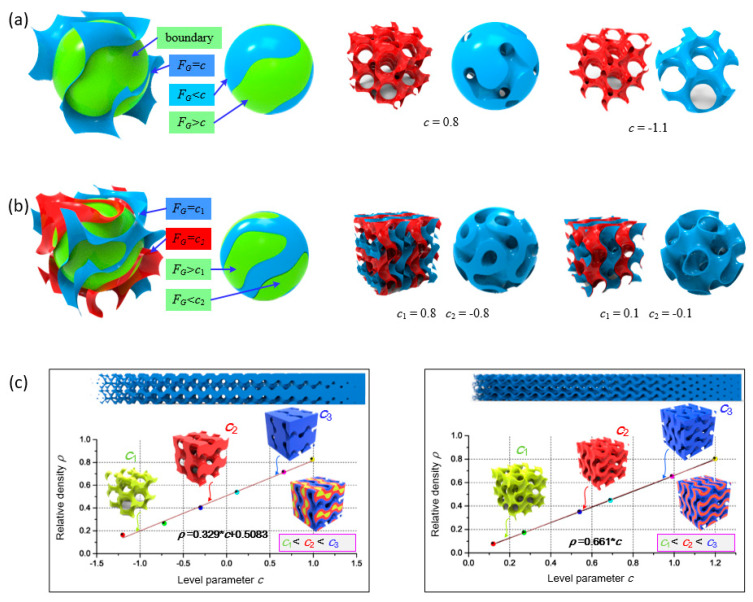
Illustration of Triply periodic minimal surface (TPMS) scaffolds: (**a**) a network-based scaffold, (**b**) a sheet-based scaffold, and (**c**) the relationship between relative density and the level parameter for two types of scaffolds.

As a type of implicit surface, a Gyroid surface can be implicitly described using the following level-set equation [52]:

(1) $$F_{G}\equiv \;\sin\left(X\right)\cos\left(Y\right)+\sin\left(Y\right)\cos\left(Z\right)+\sin\left(Z\right)\cos\left(X\right)=c$$

where *X* = 2*απx*, *Y* = 2*βπy*, and *Z* = 2*γπz*, in which *α*, *β*, and *γ* are coefficients determining the size of unit cells in the *x*, *y*, and *z* directions, respectively. In the equation, the function *F_G_* determines the surface topology, while the level parameter *c* controls the volume fraction of two domains separated by the surface. If one domain is solidly filled while the other is left empty, a network-based porous structure would be generated as shown in Figure 1a. Generally, the solid volume is defined as the domain where *F_G_* ≤ *c* for subsequent generation of porous structures together with a specific outer boundary. On the other hand, a sheet-based porous structure can be obtained using a Boolean difference operation of two TPMSs with the same topology *F_G_* but different level parameter *c*, as shown in Figure 1b.

The relative density *ρ* is an important parameter for porous materials that directly affects some mechanical properties, such as elastic modulus and yield stress [33]. For a specified Gyroid unit cell, *ρ* is directly determined by the level parameter *c* but is independent of *α, β*, and *γ*. Therefore, the relationship between parameter *c* and relative density *ρ* must be established. An approximately linear relationship can be found between them by plotting with several Gyroid samples, as shown in Figure 1c, which is consistent with previous reports [53]. On the other hand, the cell size, determined by *α*, *β*, and *γ*, can also affect several properties such as the pore size and surface area, which are important for cell adhesion and growth. The effects on the surface area exerted by cell size (determined by *α*, *β*, and *γ*) and relative density (determined by *c*) have been investigated [44].

### 2.2. Functional Gradients with TPMS

**Figure 2:**
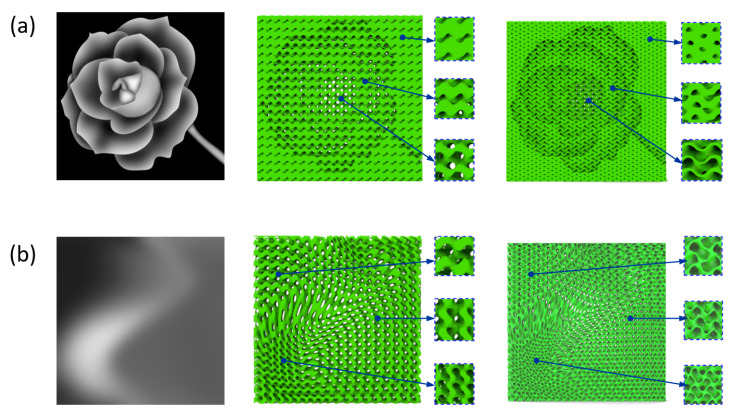
Examples of a Gyroid-based functionally graded scaffold (FGS) using network and sheet structures by (**a**) grading level parameter and (**b**) grading cell size.

FGS can be constructed by varying the level parameter or the values of *α*, *β*, and *γ* spatially depending on a certain function or tabulated data to achieve smooth variation of corresponding properties. A gradient in the relative density would appear when the level parameter *c* is designed as a continuous function in 3D space, and its mathematical function can be expressed as follows:

(2) $$F_{G}^{*}\equiv \;\sin\left(X\right)\cos\left(Y\right)+\sin\left(Y\right)\cos\left(Z\right)+\sin\left(Z\right)\cos\left(X\right)=c\left(x,y,z\right)$$

where *c* (*x*, *y*, *z*) is obtained based on the required relative density of certain points. If the relative density can be described as an s continuous spatial function to achieve complex distribution of the elastic modulus, a continuous FGS with Gyroid unit cells can be obtained. As shown in Figure 2a, a Gyroid-based FGS is generated based on the designed distribution of level parameter, which can perfectly map the expected material properties. In the example, the relative density ranges from 20% to 80% and the level parameter changes from −0.937 to 0.887 and from 0.303 to 1.21 for network and sheet-based FGS, respectively. Variation on the pore size can be achieved by varying the values of *α*, *β*, and *γ* while preserving a constant level parameter to guarantee a constant relative density. The mathematical function of the FGS with varying cell size can be expressed as follows:

(3) $$F_{G}^{\#}\equiv \;\sin\left(X^{\prime }\right)\cos\left(Y^{\prime }\right)+\sin\left(Y^{\prime }\right)\cos\left(Z^{\prime }\right)+\sin\left(Z^{\prime }\right)\cos\left(X^{\prime }\right)=c_{0}$$

where $X^{\prime }=\alpha \left(x,y,z\right)\cdot x$,$\;Y^{\prime }=\beta \left(x,y,z\right)\cdot y$, and$\;Z^{\prime }=\gamma \left(x,y,z\right)\cdot z$, in which these functions control the cell size in three directions and should satisfy some criteria to avoid shape distortion [45]. Figure 2b shows an example of the FGS constructed by the variation of cell size based on network and sheet structures, respectively.

### 2.3. FGS with Programmalbe Pore Sizes

The current strategies as described in the last section for designing FGS cannot achieve distributed pores with programmable pore sizes. Specifically, the change of level parameter with a given Gyroid unit would change the pore size to affect the overall porosity in the relative density gradient, and the pore size would also vary accordingly in the cell size gradient. The pore size distribution cannot be further tuned after the gradient design with the current methods. To satisfy more circumstances for FGS with specified pore size distribution, we design a new type of FGS by properly adjusting the relative density and cell size simultaneously to achieve programmable pore sizes. The mathematical expression of this type of FGS can be described as in Equation (4).

(4) $$F_{G}^{*\#}\equiv \;\sin\left(\;X^{\prime }\right)\cos\left(\;Y^{\prime }\right)+\sin\left(\;Y^{\prime }\right)\cos\left(\;Z^{\prime }\right)+\sin\left(\;Z^{\prime }\right)\cos\left(\;X^{\prime }\right)=c\left(x,y,z\right)$$

**Figure 3:**
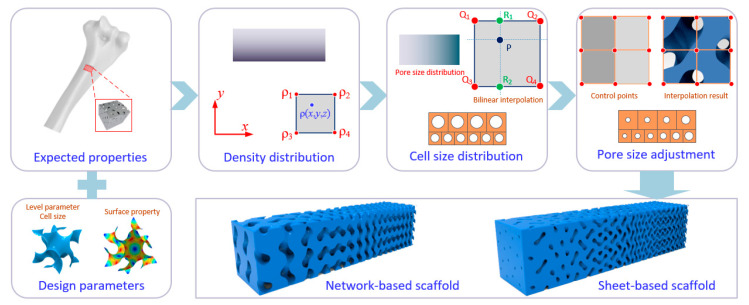
Procedure of the generation of FGS based on the expected properties with programmable pore size.

First, we need to confirm the relationship between pore size and adjustable variables, including level parameter (*c*) and coefficients determining the cell size (*α*, *β*, and *γ*). As discussed above, a negative correlation exists between the pore size and the level parameter when the cell size is specified. On the other hand, the pore size increases gradually with an increase in the cell size proportionally. Therefore, the expected properties of the target scaffold can be quantified and transferred into a 3D matrix at first to generate the density distribution for subsequent calculation of the design parameters. As the material properties of a porous scaffold are mainly affected by their relative density distribution [54], the density values for all points can be organized into a tabulated data. After that, cell size would be graded based on the calculated density matrix and the given pore size distribution using bilinear interpolation method. To achieve programmable pore sizes, the level parameter at each point *c* (*x*, *y*, *z*) is supposed to be adjusted based on the required density simultaneously. The above procedure can be described in a flowchart, as illustrated in Figure 3. Therefore, an FGS with programmable pore sizes that satisfies the required properties can be generated by simultaneous consideration of level parameter and cell size.

### 2.4. Modeling of FGS with Programmable Pore Sizes

Hence, an FGS with expected heterogeneous properties can be generated by simultaneously changing relative density and cell size, while the pore size is also programmable. The relative density is generally prior to other factors in designing FGS [55]. Based on a given 3D matrix describing the expected relative density, the porosity at each point equals to value of 1 − *ρ*(*x*, *y*, *z*). At the same time, the pore size at each point *p*(*x*, *y*, *z*) is also specified based on the intended usage, so the cell size distribution corresponding to each point *s*(*x*, *y*, *z*) can be calculated. Unlike general scaffolds with regular pores, the pore shape of Gyroid-based scaffolds is irregular and hard to be quantified using a 2D length. As illustrated in Figure 1a,b, the topology of a pore in a Gyroid unit cell is always changing, so it is reasonable to quantify the pore size by its volume within a unit cell. Therefore, *s* (*x*, *y*, *z*) can be expressed as follows:

(5) $$s\left(x,y,z\right)=\frac{p\left(x,y,z\right)}{1-\rho \left(x,y,z\right)}$$

Based on the relationship between unit cell size and coefficients, *α*, *β*, and *γ* can be readily expressed as follows:

(6) $$\alpha \left(x,y,z\right)=\beta \left(x,y,z\right)=\gamma \left(x,y,z\right)=\frac{2*\pi }{\sqrt[{3}]{s\left(x,y,z\right)}}=2*\pi *\sqrt[{3}]{\frac{1-\rho \left(x,y,z\right)}{p\left(x,y,z\right)}}$$

Therefore, the coefficients (*α*, *β*, and *γ*) in the implicit function at each point can be obtained based on Equation (6), and the level parameter *c* (*x*, *y*, *z*) is simultaneously determined according to Equation (7).

(7) $$c\left(x,y,z\right)=H^{-1}\left[\rho \left(x,y,z\right)\right]=\{\begin{array}{c} \frac{\rho \left(x,y,z\right)-0.5083}{0.329}\;\;\;network\;structure\\ \frac{\rho \left(x,y,z\right)}{0.661}\;\;\;\;\;\;\;\;\;sheet\;structure \end{array}$$

where *H* is the function defining the relationship between the level parameter and relative density and can be obtained from Figure 1c.

## 3. Fabrication and Characterization

### 3.1. Model Preparation for Fabrication

The topology and volume fraction of Gyroid-based structures are totally controlled by the level parameter and cell size based on Equation (4). When the level parameter and cell size have been defined in a 3D data field, the solid model can be created by extracting the surfaces using the Marching Cubes (MC) algorithm [56]. After that, triangular facets are obtained and saved as STL (Stereo Lithography) models. Here, Gyroid-based structures with functional gradient were generated using *C#* codes by defining the equations of TPMS surfaces as well as by providing the Boolean operations between the defined surfaces and the specified outer boundary (a cube in this work). It should be noted that a challenge in using the MC algorithm for surface extraction is achieving a balance between high accuracy and a proper file size. As a result, the transition area between the TPMS surface and the outer boundary has some sharp geometries which are not beneficial for fabrication, so the Magics software (Materialise, Leuven, Belgium) was used to repair these geometries.

Four different FGSs were studied, as illustrated in Table 1. A linear gradient in the relative density from 0.4 to 0.2 along the Z axis was assigned for all samples. The side length of samples was 15 mm. An FGS with constant pore size at the value of 18.9 mm^3^ was chosen in the first two groups as a typical FGS with programmable pore sizes for investigation. Based on Equations (5)–(7), the coefficients (*α*, *β*, and *γ*) varied from 1.988 to 2.189, while the level parameter changed from *H*^−1^(0.4) to *H*^−1^(0.2) accordingly. The FGSs with constant pore size based on network (NP) and sheet structures (SP) were generated using these parameters. The void parts were also rendered for better visualization. It can be observed that porosity and interconnectivity were achieved with the proposed method. The pore size (represented by a green color) was kept constant based on the design intension, while the relative density and the cell size were linearly changed, as can be seen from the side view. For comparison, another two groups of general FGS with constant cell size (NC and SC) were also generated. The coefficients were set as 2.088 to obtain constant and similar unit cell size with the edge length of 3 mm. As can be seen, a continuous gradient in the relative density was obtained and the unit cell size was always the same within the whole structure. The pore size in the latter two groups was varied as the factor determining the relative density. The nominal average relative density of samples can be calculated by the volume fraction of solid parts.

### 3.2. SLM Fabrication and Visual Characterization

All samples were fabricated using the DiMetal-100 SLM machine (LeiJia, Guangzhou, China) with Ti6Al4V ELI powder (Oerlikon Metco Inc., Westbury, NY, USA), and a 150-W fiber laser with a beam diameter of 60–80 μm was assembled. The layer thickness was set to 30 μm for adequate accuracy. The particle size distribution of the powder was d10 = 21.0 μm, d50 = 34.0 μm, and d90 = 46.0 μm, and the chemical composition of the powder is listed in Table 2 provided by the supplier. All fabricated samples were carefully cleaned through a sandblasting posttreatment to remove adhered powder particles using a sandblasting machine.

A digital microscope (Axio imager A2m, Zeiss, Oberkochen, Germany) was used to capture microscopic images of additively fabricated samples, especially focusing on the surface morphologies of samples in order to evaluate their manufacturability. The overall density of samples was calculated by dividing their masses by the mass of a solid cylinder with the same dimensions. The measured mass might be slightly larger due to adhered particles inside the fabricated specimens. The mass was weighed using an electronic balance, and dimensions were measured using the digital micrometer to calculate the volume. The surface area was approximately evaluated based on the models.

### 3.3. Investigation of Mechanical Properties

For investigating the mechanical properties, uniaxial compressive tests were conducted on a MTS testing machine (Instron, Shanghai, China) equipped with a maximal load capacity of 200 KN. The FGS samples were compressed under displacement-controlled conditions with a loading rate of 2 mm/min, accompanied by a video camera capturing the deformation behaviors. The applied loading direction aligns with the printing direction for all samples. The compression process was terminated when the loading displacement reached at a certain value (8 mm) considering the maximal strain. The record force and displacement for each sample would be used to obtain the corresponding stress–strain curves considering the height and cross-sectional area of samples. The Young’s modulus was calculated by the slope in the elastic stage, and the yield stress was determined considering a strain offset of 0.2%. The plateau stress was the average value of the stresses between 0.2 and 0.5 of the strain.

## 4. Results and Discussion

### 4.1. Microstructural Characterization

The additively manufactured FGS samples of each group were weighted and then used to calculate their actual relative densities. The average masses and corresponding deviations for each group (5 replicas) are listed in Table 3, and the bulk density was 4.52 g/cm^3^ based on preliminary studies on fabricated specimens. Hence, their actual relative densities were calculated by dividing the mass. Deviations in the relative densities (RD) between fabricated samples and designed CAD models existed, as can be observed. The reasons behind the deviation can be attributed to the technical limitations of selective laser melting (SLM). The powder particles close to the boundary would adhere to the fabricated parts due to the thermal difference between unmolten and molten powder [45]. At the same time, the molten particles along the tracking paths are not heated evenly; partial melting surrounding the edge of the paths would appear, resulting in more bonded powders. Moreover, there are many small geometric features in the designed FGS models causing the bonded particles to be not easy to clean after fabrication. That is why the error of sheet-based scaffolds is larger than that of network-based ones. All of these mentioned process-related factors can result in excess of the actual RD over designed models.

**Figure 4:**
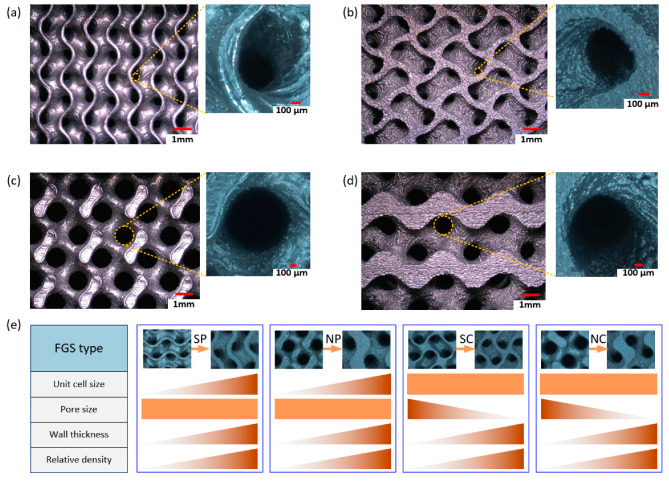
Microstructural analysis of different types of FGS: (**a**) top surface of SP, (**b**) bottom surface of SP, (**c**) top surface of NP, (**d**) bottom surface of NP, and (**e**) changes in related parameters with grading relative density.

Figure 4 shows the captured surfaces of the fabricated samples. As for the types of SP and NP, the change in relative density was achieved by simultaneously controlling the level parameter and unit cell size to confirm programmable pore size. The pore sizes on the top surface (Figure 4a,c) and the bottom surface (Figure 4b,d) of the fabricated FGS samples were supposed to be the same. On the other hand, the pore sizes were always changing along the grading direction to confirm constant unit cell size for the types of SC and NC. The pore size and wall thickness could be measured from the captured pictures, and their changes along the grading direction are illustrated in Figure 4e. Take SP as an example, the designed relative density at the bottom surface was 0.4, the edge length of a unit cell was 3.43 mm, while it decreased to 2.57 mm on the top surface with a decreased relative density of 0.2. The wall thickness was affected by the level parameter as well as the unit cell size. The measured wall thickness at the bottom surface is about 0.32 mm, while it reduces to 0.12 mm on the top. As illustrated in Figure 4e, both SP and NP could achieve uniform pore size within the whole scaffold by varying the wall thickness and unit cell size at the same time. The wall thickness changed with the same trend as the unit cell size to make sure that the pore size unchanged. However, the unit cell size was always the same in SC and NC, the pore size decreased in company with the increasing wall thickness based on the expected distribution of relative density.

**Figure 5:**
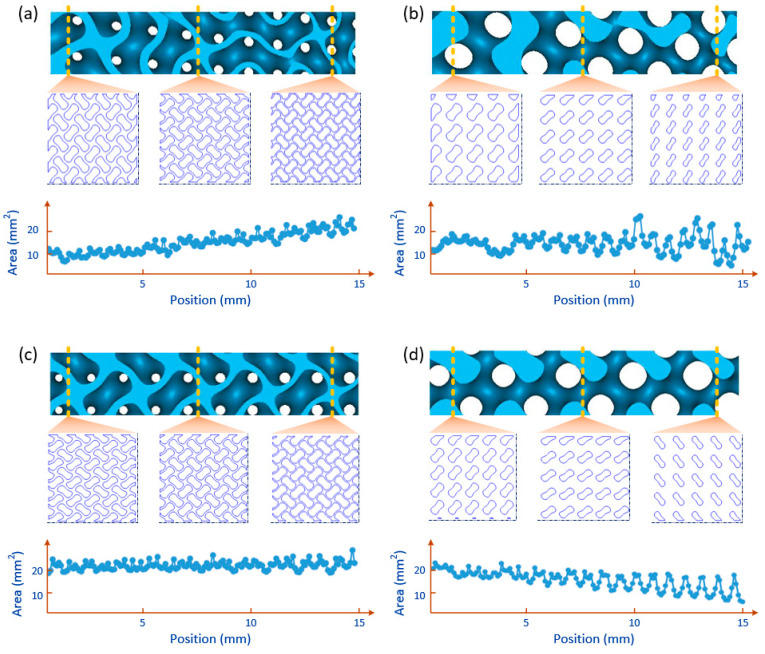
Analysis and comparison of surface area for different types of FGS based on a slice method: (**a**) SP, (**b**) NP, (**c**) SC, and (**d**) NC.

Surface area plays an important role on cell adhesion for scaffolds, and its gradient caused by the design parameters was discussed here. From a three-dimensional perspective, the surface area of a scaffold can be calculated by counting all the small surfaces, but it is difficult to figure out the change in surface area along a certain direction. In this study, the 3D model was sliced into layers along the grading direction and the surface area of each layer could be approximately obtained using the product of the contour length and the layer height. By doing so, the effect on the surface area of design parameters could be investigated. As illustrated in Figure 5, the CAD models were sliced with a layer height of 0.1 mm and the contour length of each layer could be obtained. As for SP in Figure 5a, the surface area slightly increased because the unit cell size gradually went down when the relative density varied from 0.4 to 0.2, while the only change of level parameter could barely affect the surface area for the sheet-based scaffold as seen from SC in Figure 5c. However, the decreasing level parameter in NC could result in a smaller surface area due to the shrinking struts in the scaffold, as shown in Figure 5d. The combined effects of decreasing unit cell size and decreasing level parameter for NP enabled the surface area to not change monotonically, as observed from Figure 5b. It should be noted that the above discussion was conducted on the basis of the relative density ranging from 0.4 to 0.2.

### 4.2. Mechanical Properties

The mechanical properties of manufactured FGSs were characterized by compression tests. All samples were graded from 0.2 to 0.4 in relative density, and the loading was applied to the grading direction. Three samples were tested for each design, and their compressive responses were nearly matched, verifying the high repeatability of the adopted SLM process.

**Figure 6:**
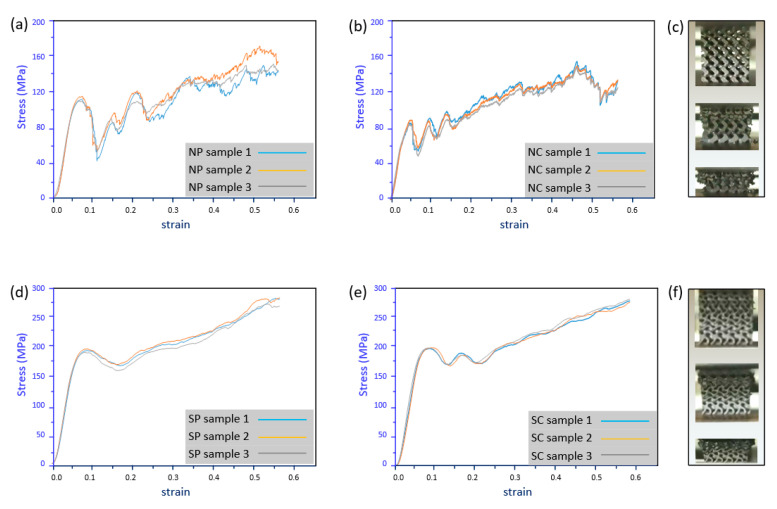
Stress–strain curves of (**a**) NP, (**b**) NC, (**d**) SP, and (**e**) SC and deformation response of (**c**) network-based and (**f**) sheet-based structures in compressive tests.

The experimental stress–strain curves of the network-based FGS with constant pore size (NP) and constant cell size (NC) are shown in Figure 6a,b. An initial nonlinear stage appeared due to the rough and uneven surface of the sample before establishing full contact [50]. The curves continued with a linear elastic state, where the elastic modulus of the structure could be determined from the curve slope. After that, the stress climbs up to the yield strength and the elastic-plastic stage appeared at about 0.08 strain. An abrupt collapse of the stress appeared due to a loss of strength, which is a common brittle failure behavior of strut cellular materials [45]. Then, a layer-by-layer deformation process took place and the stress–strain curves experienced several peaks and valleys with an upward trend. For network-based structures, the stress concentration on the struts during the compression process as well as the brittle fracture would result in some crumbled fragments. It could be observed that the stress–strain curves of specimens in each group matched well in the early stage while the discrepancy became significant gradually, which could explain the crushed fragments being located randomly inside the scaffolds and affecting the volume distribution of the remaining structures. From Figure 6c, the collapse happened from top to bottom, so the early stage was mainly affected by the upper part. Compared to the NC type, the cell size was smaller and the cell number was larger in NP, so its yield strength was relatively higher. The deformation continued downwards to the bottom layers, and the crushed parts were stacked and started to contact the next layer.

Figure 6d,e illustrates the stress–strain curves of sheet-based FGS with constant pore size (SP) and constant cell size (SC). Compared to network-based FGS, the curves for both SP and SC were highly matched since the sheet-based structures during the compression process would not be crumbled. Similarly, the elastic stage finished at around 0.08 strain and the stress dropped with a slightly smooth trend. Then, the curve started to increase gradually with a non-obvious layer-by-layer response because the change of the cross-sectional shape between layers was much smaller, as illustrated in Figure 5. Additionally, the deformation behavior as shown in Figure 6f indicates that the collapse moved slightly downwards and that the deformed parts densified and enabled the top layers to become much harder.

Performance based on the experimentally obtained stress–strain curves, such as Young’s modulus (*E*), yield strength (*σ*_s_), ultimate strength (*σ*_max_), and plateau stress (*σ*_pl_), are summarized in Table 4. It shows that all mechanical properties of the sheet-based FGSs were significantly higher than that of network-based FGSs. This could be explained by the sheet structures possessing smaller pores and much thinner walls, which would buckle under compression. The buckled walls resulted in a gentler stress collapse after each peak and less stress fluctuation in the plateau stage. On the other hand, the Young’s modulus and yield strength of NP were slightly higher than that of NC due to the smaller cell size on the top layers [44]. Besides the process-related errors mentioned in Section 4.1, the inconsistency during the compression tests would also affect the measured data. These factors can explain the experimentally obtained deviation of mechanical properties between different specimens. Based on the Gibson–Ashby plot, their performance with respect to other engineering materials can be specified [57]. It can be concluded that all the designed graded porous structures fall into the domain of natural materials based on their densities and Young’s moduli. Therefore, the porous structures with the material can be adopted in the field of bone scaffolds.

### 4.3. Discussion

Scaffold design based on implicit function has been proven to be versatile, as it allows geometries to be simply designed by pure mathematical expressions. There exist variables in the equations affecting the relative density and unit cell size, which are essential to controlling the biomechanical properties of the fabricated TPMS scaffolds [57]. Therefore, parametric design of scaffolds for structural gradients can be realized by properly tuning related parameters. On the other hand, the design of scaffolds for bone tissue engineering should be guided by different structural and functional material properties, such as adequate porosity and multi-scale organization and hierarchy [58,59]. Different strategies, such as gradients in density, cell size, or cell shape, have been proposed to satisfy the required specific properties and architectures. However, the pore size varies without any quantitative control in these gradients and its relationship with all the design parameters has not been comprehensively considered yet.

**Figure 7:**
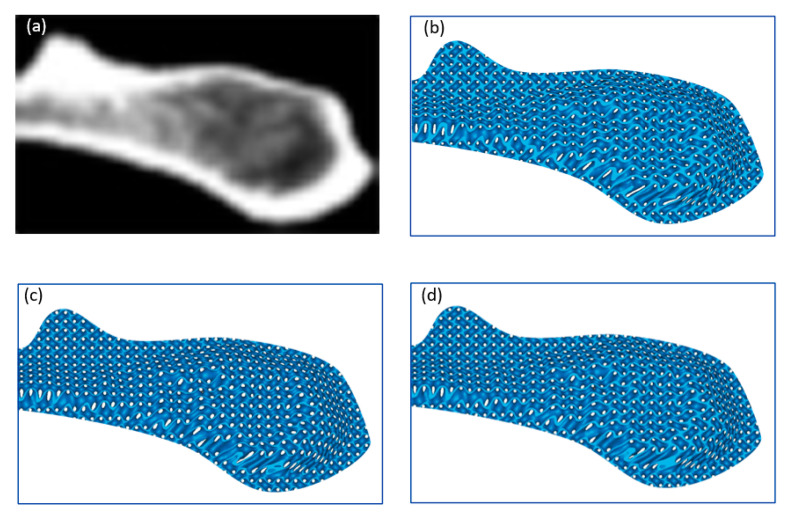
A generated FGS with programmable pore size using two methods: (**a**) a CT image of a bone tissue, (**b**) a constructed FGS based on the specified relative density, (**c**) a generated FGS with varying unit cell size and constant level parameters based on the expected unit cell size distribution, and (**d**) adjusted level parameters to obtain a programmable pore size based on (**c**).

The pore size of TPMS scaffolds is controlled by both the unit cell size and relative density. Changing either of the two parameters would result in variation in pore size. To obtain a functional gradient with programmable pore size, both of them must to be adjusted based on the expected properties. The strategy illustrated in Figure 4 is proposed based on the premise that the expected properties are described by the density distribution, so the unit cell size is accordingly adjusted spatially. However, there are some circumstances where the unit cell size has the highest priority while the relative density is allowed to be changed. The proposed method can also be adapted to satisfy this requirement. The specified distribution of the unit cell size is achieved by determining the coefficients at each point within a given domain, followed by adjustment of the level parameter based on the designed pore size distribution. Figure 7 shows an example of the generated sheet-based FGS with programmable pore size based on the specified relative density and unit cell size, respectively. In the former method, the unit cell size distribution is directly determined by the relative density and pore size, while it is specified in advance and the relative density (controlled by level parameters) needs to be adjusted to achieve a programmable pore size in the latter method.

Therefore, the pore size distribution is fully programmable in the proposed method. The pore size distribution can be provided as a 3D matrix based on the functional gradients of natural bone tissues. At the same time, the distribution of relative density or unit cell size is specified based on the expected properties. Then, the FGS with two expected gradients can be achieved. When the pore size and relative density are specified, the unit cell size at each point can be calculated based on Equation (5). If the pore size and unit cell size are given, the level parameter at each point can be determined based on Equation (7).

Both network and sheet FGS with programmable pore size can be successfully additively fabricated using SLM. Their mechanical performance shows good agreement with that of FGS constructed by grading level parameters, indicating the potential for mimicking bones in terms of morphology and mechanical properties. A sheet FGS possesses superior performance of mechanical behavior and larger surface area compared with a network FGS, but its pore size is much smaller under the same relative density. The attached powder particles inside the pores are very difficult to remove, and this is verified by the actual relative density of fabricated specimens, as illustrated in Table 3. Moreover, the wall thickness of sheet FGS is much narrower, which requires consideration on the limitations of SLM fabrication process. Therefore, the SLM processing parameters should be integrated into the design process to ensure successful manufacturing.

## 5. Conclusions

A new type of TPMS-based FGS with programmable pore size distribution was parametrically designed, and the difference on the structural morphologies and mechanical properties compared to general FGSs was investigated. TPMS was verified as an effective and feasible tool for designing porous and interconnected structures with desired functional gradients in terms of relative density and unit cell size. The pore size was affected by the cell size and level parameter, both of which could be effectively controlled by tuning parameters in the mathematical equation while maintaining smooth transition. Both network and sheet-based FGSs with the proposed method were designed and fabricated by SLM. The geometric morphologies of specimens, including pore size, wall thickness, and surface area, were studied from the microscopic observations and designed models. The results showed that the fabricated FGSs with programmable pore sizes could achieve expected gradients with the mapping method. In terms of mechanical properties, the compression tests were performed and the obtained stress–strain curves were compared. A layer-by-layer collapse could be observed from the compression process due to the structural characteristic of TMPS. Under the same porosity gradient, the graded structures with programmable pore sizes did not significantly affect the mechanical performance compared to FGS with constant cell sizes. The sheet-based FGS showed superior mechanical properties, not only in Young’s modulus and yield strength but also with less stress fluctuation during the compression process.

TPMS structures with programmable pore sizes have the potential to create an FGS for applications where pore size has the highest priority and can accompany other grading requirements on morphology and mechanical properties. Besides bone scaffolds, the proposed structures can also be adopted in designing and optimizing lattice structures with high load-bearing and energy absorption capacities. The gradients in pore size are widely found in actual bone tissues and can be effectively generated using the proposed strategy to further investigate their effects on bone regeneration. In addition, the fatigue properties of additively fabricated porous structures are essential for long-term use [22], so the fatigue behavior of the proposed structures and their underlying fatigue mechanism should be further studied by combining high-cycle compression–compression fatigue testing and FEA (finite element analysis).

## Figures and Tables

**Table 1:** Analysis and comparison of a sheet/network-based FGS with programmable pore sizes and constant cell size: (**a**) sheet-based FGS with programmable pore size (SP), (**b**) network-based FGS with programmable pore size (NP), (**c**) sheet-based FGS with constant cell size (SC), and (**d**) network-based FGS with constant cell size (NC).

Type	Solid Part	Void Part	Combined Structure	Pore Distribution	Fabricated Part
**(a) SP**	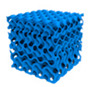	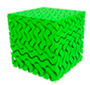	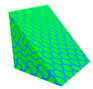	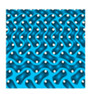	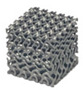
**(b) NP**	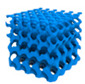	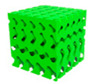	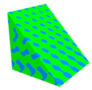	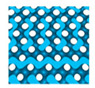	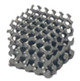
**(c) SC**	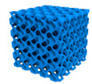	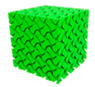	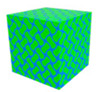	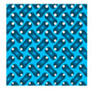	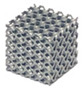
**(d) NC**	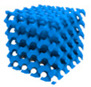	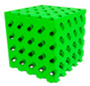	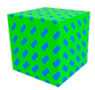	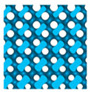	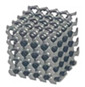

**Table 2:** Chemical analysis of the Ti6Al4V ELI powder.

Element	Al	C	Fe	Ti	V	T.A.O.	H	O
**wt%**	6.39	0.02	0.16	89.33	3.95	0.05	0.002	0.10

**Table 3:** Relative densities (RD) of fabricated samples.

Type	Grading Density	Measured Mass (g)	Theoretical RD	Actual RD	Error
**SP**	0.2–0.4	4.75 ± 0.16	0.2936	0.3113	6.0%
**NP**	0.2–0.4	4.62 ± 0.27	0.2945	0.3028	2.8%
**SC**	0.2–0.4	4.78 ± 0.14	0.2951	0.3133	6.1%
**NC**	0.2–0.4	4.58 ± 0.19	0.293	0.3002	2.5%

**Table 4:** Mechanical properties of different types of FGS.

Type	E (GPa)	*σ*_s_ (MPa)	*σ*_max_ (MPa)	*σ*_pl_ (MPa)
**NP**	3.16 ± 0.05	98.58 ± 3.34	109.67 ± 2.73	122.99 ± 5.81
**NC**	2.58 ± 0.17	73.43 ± 2.16	90.52 ± 7.50	114.18 ± 2.77
**SP**	4.32 ± 0.13	179.17 ± 0.15	195.57 ± 1.47	227.92 ± 3.03
**SC**	4.31 ± 0.09	178.17 ± 0.11	187.03 ± 2.63	228.54 ± 0.43
